# Using nominal group technique to select an HIV status disclosure decision aid for adaptation in Georgia

**DOI:** 10.1371/journal.pone.0353949

**Published:** 2026-07-16

**Authors:** Tamar Zurashvili, Mariam Pashalishvili, Akaki Abutidze, Fiona Evelene, Nino Chikhladze, Mamuka Djibuti

**Affiliations:** 1 Faculty of Medicine, Ivane Javakhishvili Tbilisi State University, Tbilisi, Georgia; 2 Partnership for Research and Action for Health, Tbilisi, Georgia; 3 National Center for Disease Control and Public Health of Georgia, Tbilisi, Georgia; 4 T. Tsertsvadze Infectious Diseases, AIDS and Clinical Immunology Research Center, Tbilisi, Georgia; 5 University at Albany, State University of New York, Albany, New York, United States of America; Fudan University, CHINA

## Abstract

**Introduction:**

HIV status disclosure decision-making is a complex process influenced by stigma, relationship dynamics, and anticipated social consequences. Although decision aids can support individuals in navigating such decisions, no disclosure decision-support interventions have been adapted for use in the Georgian context. This study aimed to identify and prioritize an existing evidence-based intervention for adaptation to support HIV disclosure decision-making among people living with HIV (PLWH) in Georgia.

**Methods:**

We used the Nominal Group Technique (NGT), a structured consensus method, to elicit and prioritize stakeholder perspectives. Two separate NGT sessions were conducted with HIV care providers (n = 12) and PLWH (n = 10), followed by a joint session to reach consensus. Prior to the sessions, nine evidence-based disclosure decision-support interventions from HIV, mental health, and substance use fields were identified through a desk review and grouped into session-based, paper-based, and digital formats. Participants generated ideas, discussed advantages and limitations, and ranked intervention formats and specific interventions. Descriptive content analysis was used to summarize discussion themes.

**Results:**

Providers prioritized digital interventions, emphasizing accessibility and scalability, whereas PLWH preferred session-based interventions, highlighting the importance of trust, individualized support, and peer involvement. Within these formats, providers favored a structured digital program, while PLWH selected an individual session-based intervention focused on disclosure to family members. Adaptation priorities included incorporating peer educators, addressing disclosure to different social and healthcare contexts, and including locally relevant content on legal issues, treatment adherence, and available support services. In the joint session, consensus was reached to prioritize the intervention selected by PLWH.

**Conclusions:**

This study identified a priority disclosure decision-support intervention for adaptation to the Georgian context, emphasizing the importance of patient-centered and contextually tailored approaches. Future research will focus on adapting the selected intervention and identifying appropriate implementation strategies to support pilot testing and integration into HIV care services.

## Introduction

HIV status disclosure decision-making is widely recognized as a complex and dynamic process shaped by stigma, relationship factors, anticipated social consequences, safety concerns, and access to support [1–5]. Disclosure may provide important benefits, including increased emotional and social support, improved communication with partners and family members, and, in some settings, better engagement in HIV care and treatment [6–8]. At the same time, disclosure can expose individuals to stigma, discrimination, blame, rejection, breaches of confidentiality, and in some cases intimate partner violence, making disclosure unsafe or undesirable for some people [9–12]. HIV disclosure decisions may also vary depending on the intended recipient of disclosure, such as sexual partners, family members, friends, or healthcare providers, each of which may involve different considerations and potential consequences.

While the importance of HIV status disclosure may be perceived as reduced in the era of “Undetectable = Untransmittable” (U = U), it remains highly relevant from a public health perspective. The preventive benefits of viral suppression depend on consistent adherence to antiretroviral therapy (ART), and interruptions in treatment can compromise viral suppression and increase the risk of onward transmission [13,14]. In this context, HIV status disclosure may remain particularly important in settings where sustained treatment adherence is not fully achieved. Evidence from Georgia shows that 23.0% of PLWH reported ever interrupting or stopping treatment, and among those, 35.3% reported not disclosing their HIV status to their sexual partner, suggesting that non-disclosure may co-occur with interruptions in ART [2].

Because HIV status disclosure involves weighing potential risks and benefits within complex social contexts, many people living with HIV (PLWH) require structured support when making disclosure decisions. Patient decision aids (DAs) are structured tools designed to support individuals facing complex health decisions by providing evidence-based information about available options and helping users clarify personal values and preferences related to those options [15]. Disclosure decision-support interventions can help individuals evaluate the potential advantages and disadvantages of disclosure, clarify personal values, and develop strategies for deciding whether, when, and to whom disclosure may be appropriate. Evidence from research on stigmatized conditions such as HIV and mental illness shows that disclosure DAs can help individuals clarify their needs and values, reduce uncertainty, and support more informed and confident decision-making [16,17].

In Georgia, recent findings similarly suggest that HIV disclosure is shaped by stigma, prior disclosure experiences, and concerns about potential social consequences, underscoring the need for interventions that help PLWH make informed and safe disclosure decisions [2]. However, despite the growing recognition of the importance of disclosure decision-making, there are currently no disclosure decision-support interventions that have been specifically adapted to the Georgian context. Thus, the objective of this study was to use the Nominal Group Technique (NGT), a structured group consensus method that combines idea generation, discussion, and ranking [18,19], to prioritize an existing disclosure DA intervention from the HIV and mental health fields that could potentially be adapted to support HIV status disclosure decision-making among PLWH in Georgia.

## Methods

### Study design and setting

This study used the NGT to identify and prioritize a disclosure DA intervention, based on existing interventions described in the literature, for adaptation to the Georgian context. NGT is a structured consensus method that supports balanced participation and is commonly used in health research to generate ideas, discuss priorities, and rank options within a group [18,19]. Its core stages include silent idea generation, round-robin sharing, clarification of ideas, and independent ranking. This study used the following approach: first we conducted two sessions: a NGT session with HIV care providers and a NGT session with PLWH, followed by a final joint session to review findings from the two separate groups and reach consensus on the intervention to be adapted. This multi-group application of NGT is consistent with its flexible use in health research, including examples where separate stakeholder perspectives are elicited and subsequently integrated through additional group discussion and consensus processes [18].

All sessions took place at T. Tsertsvadze Infectious Diseases, AIDS and Clinical Immunology Research Center in Tbilisi (AIDS Center), the capital of Georgia. The center is the main national institution responsible for the development, coordination, and implementation of activities related to HIV/AIDS prevention, diagnosis, treatment, and research in Georgia.

### Participant recruitment

Participants were recruited separately for the provider and PLWH sessions from February 9 to March 3 2026. HIV care providers were recruited at the AIDS Center. A research focal point at the AIDS center who was responsible for providers’ recruitment for the NGT session approached potential participants who were involved in HIV care and support services, briefly explained the purpose of the study, obtained oral agreement to participate, and invited interested individuals to attend the scheduled NGT session. PLWH were recruited through the community-based organization Real People Real Vision, a PLWH-led organization working with the HIV community in Georgia. A community leader from the organization approached potential participants, explained the study, obtained oral agreement to participate, and invited them to attend the scheduled NGT session. Written informed consent was obtained from all participants before the start of the NGT session. To protect participant confidentiality, informed consent forms were stored separately from session materials and were not linked to ranking forms or discussion notes. Participants who preferred not to sign the consent form themselves were offered the option of having their consent documented by the recruiter, who signed the consent form on their behalf in accordance with the IRB-approved consent procedures. No personal identifiers were recorded in the discussion notes. All notes and ranking data were de-identified prior to analysis.

### Identification and preparation of candidate interventions

Prior to the NGT sessions, a desk review was conducted to identify existing interventions designed to support disclosure decision-making. Interventions were selected based on predefined criteria, including evidence of effectiveness (e.g., randomized controlled trials or pilot evaluations), relevance to disclosure decision-making in stigmatized conditions (such as HIV, mental health, substance use), and feasibility for adaptation to the Georgian context. Particular consideration was given to interventions that are low-cost, scalable, and can be delivered within routine service settings. A total of nine interventions meeting these criteria were identified and grouped into three categories based on their primary format: session-based interventions, paper-based interventions, and digital interventions.

#### Session-based interventions.

The following three interventions were identified within session-based interventions. (1) The Honest, Open, Proud (HOP) intervention is a group-based program originally developed for individuals living with mental health conditions. The intervention typically consists of three group sessions of approximately two hours each, delivered weekly by a trained peer, followed by a booster session approximately one month later. The sessions guide participants through structured reflection on the potential benefits and risks of disclosure, the different levels of disclosure, and strategies for assessing whether disclosure may be appropriate in a particular context. Participants also practice how to share their personal story and discuss the role of peer support. Evidence suggests that HOP can reduce self-stigma, decrease stress related to disclosure decisions, and improve individuals’ confidence in making disclosure decisions [20]. (2) The Positive, Open, Proud (POP) intervention was adapted specifically for PLWH, particularly women. Similar to HOP, POP consists of three structured group sessions followed by a booster session. The program supports participants in weighing the advantages and disadvantages of disclosure, deciding whether and how to disclose their HIV status, and developing a personal narrative for sharing their experience with others. Preliminary evidence suggests that integrating elements of the HOP intervention into POP may help reduce HIV-related stigma [21]. (3) A decision-making intervention to help decide whether to disclose HIV-positive status to family members (Family disclosure DA intervention) consists of seven individual sessions lasting approximately 50–90 minutes each and delivered over a period of about twelve months. The sessions focus on helping individuals carefully consider the potential risks and benefits of disclosure, plan disclosure strategies, anticipate possible reactions from family members, and develop communication skills through role-playing exercises. Although research did not demonstrate a statistically significant effect on disclosure behavior itself, the intervention showed positive effects on participants’ well-being and sexual health outcomes, suggesting that structured decision-making support may still provide meaningful benefits [17].

#### Paper-based interventions.

Similarly, three interventions were identified within the paper-based intervention category. (1) The CORAL DA is a printed booklet developed to support individuals with mental health conditions who are considering whether to disclose their condition in the workplace. The booklet typically contains approximately 12–13 pages and includes structured sections that guide users through reflection on the potential advantages and disadvantages of disclosure, personal needs and values, appropriate timing for disclosure, and decisions about whom to inform. The booklet concludes with a section that helps individuals summarize their reflections and decide whether disclosure is appropriate in their situation. Studies evaluating CORAL have found that the intervention can reduce decision-related conflict among participants compared with control conditions [16]. (2) An adapted version of the CORAL**,** CORAL.NL**,** was developed for individuals with mental health conditions seeking employment in the Netherlands. The intervention consists of a 14-page booklet organized into four main sections that guide users through reflection on disclosure choices, personal circumstances, and planning of disclosure strategies. To improve accessibility for individuals who may experience reading or concentration difficulties, the intervention also includes two one-page infographics summarizing the benefits and risks of disclosure and providing practical guidance for disclosure decisions during job applications and employment. A randomized controlled trial found that the intervention improved employment outcomes, with significantly more participants finding and retaining work compared with the control group [22]. (3) Another intervention in this category was Disclosing Recovery, a structured disclosure DA developed for individuals receiving opioid substitution therapy. The intervention is delivered through a printed workbook and worksheet and is typically conducted in a structured session lasting approximately one hour. The intervention also includes a skills-building component in which participants develop a disclosure script and a concrete plan outlining when, how, and to whom they may disclose their recovery status. A pilot randomized controlled trial found that the intervention improved the quality of disclosure decision-making and was considered acceptable and feasible by participants [23].

#### Digital interventions.

Three interventions were identified within the digital intervention category. (1) The READY intervention is a self-guided digital program consisting of seven short interactive modules, each lasting approximately five to ten minutes. The program was developed for employed individuals with mental health conditions who are considering whether to disclose their condition in the workplace. The modules guide participants through reflection on the potential consequences of disclosure and non-disclosure, personal values and needs, and the timing and process of disclosure. The intervention also includes interactive summaries that help participants synthesize their responses and clarify their decision. In a randomized controlled trial, the intervention significantly reduced decisional conflict and improved participants’ satisfaction with their disclosure decision compared with the control group [24]. (2) The DISCLOSURE intervention is a digital DA designed for adolescents and adults with autism spectrum conditions. The intervention consists of three interactive PDF documents combined with video materials and reflective exercises. The first two documents provide information about disclosure concepts, workplace characteristics, personal needs, and identity considerations. The third document includes checklists and reflective questions that help participants evaluate potential disclosure targets and workplace factors. A pilot study showed that the program was acceptable and supported autistic adults in making more informed decisions about disclosure of their diagnosis [25]. (3) The final intervention identified was “Who, When, How to Share”, a digital decision-support guide adapted from the HOP intervention. The intervention consists of a detailed 84-page guide accompanied by seven worksheets that help users analyze disclosure decisions step by step. The guide is organized into sections addressing when to disclose, how to disclose, and to whom disclosure may occur. The materials also include information on legal rights and available support services. The intervention is typically completed over several weeks, with each section requiring approximately 60–90 minutes of engagement. A pilot study suggested that the intervention was acceptable and may help reduce decisional conflict and disclosure-related distress [26].

### NGT procedure

For the NGT sessions, the research team prepared one-page summaries of the nine interventions described above, including information on their target population, format, duration, main components, and available evidence. Both the provider and PLWH sessions followed the standard NGT procedure and each lasted approximately 60 minutes. At the beginning of each session, participants received printed copies of the intervention summaries, and the moderator briefly presented each intervention and its format. Participants were given time to review the materials and ask clarification questions. Participants were also provided with standardized verbal instructions on the ranking process. They were asked to consider multiple factors when evaluating and ranking the interventions, including their perceived appropriateness for PLWH in Georgia, potential acceptability to end users, feasibility of implementation within the Georgian healthcare context, scalability, and overall potential to reduce HIV-related stigma. For the first exercise, participants were asked to discuss the advantages and disadvantages of the three intervention formats (session-based, paper-based, and digital) for use in the Georgian context. Participants first generated ideas individually and silently. This was followed by a round-robin sharing process, during which participants presented their ideas one at a time. A rapporteur recorded all ideas on a flip chart without discussion or critique. After all ideas had been listed, the group engaged in a structured discussion to clarify and elaborate on the points raised. Participants then independently ranked the intervention formats. Each participant was given three points that could be allocated across the options according to their preference. Participants could assign all three points to a single option, distribute one point to each of the three options, or allocate two points to one option and one point to another. The points assigned by all participants were then aggregated to produce a prioritized list of intervention formats. Following the identification of the preferred intervention format in each group, a second ranking exercise was conducted to compare the interventions within that format (in the provider session, participants compared the three digital interventions, while in the PLWH session participants compared the three session-based interventions). Participants discussed the strengths and limitations of each intervention and then independently ranked them using the same scoring procedure as in the first exercise. In the final part of each session, participants were asked what adaptations would be necessary to make the highest-ranked intervention appropriate for the Georgian context of PLWH. This part of the discussion was conducted as an open brainstorming exercise rather than a formal ranking process. After the two separate NGT sessions, a 40-minute joint session was conducted with representatives from both groups. During this session, the facilitator presented a summary of the findings from the provider and PLWH sessions, and participants discussed similarities and differences between the two groups’ perspectives. The discussion aimed to reach consensus on the intervention that would be prioritized for adaptation. Consensus was reached through facilitated group discussion rather than a formal consensus method. Consensus was defined as general agreement among participants regarding the intervention to be prioritized for adaptation. Although providers and PLWH expressed different viewpoints regarding the intervention selection, no major disagreements emerged during the joint session. Minor differences in opinion were addressed through facilitated discussion, allowing participants to clarify their perspectives and consider the views of others before reaching a shared decision. The final selection process was designed to prioritize the perspectives of PLWH as the primary end users of the intervention, while also incorporating provider views on feasibility and implementation. Each NGT session was conducted as a single plenary group. Participants were not divided into smaller groups at any stage of the process, and all idea generation, discussion, ranking, and consensus activities were completed with the full group present. To enhance the credibility of the qualitative findings, discussion notes were recorded by a designated rapporteur during each session and were reviewed by members of the research team immediately after the sessions to ensure completeness and accuracy.

### Data analysis

Data generated during the NGT sessions included ideas recorded on flip charts during the discussions and the ranking scores assigned by participants (S1 File). Ranking scores were aggregated to identify the preferred intervention format and the preferred intervention within each format. Discussion notes from the NGT sessions (S2 File) were reviewed using a descriptive content analysis approach [27]. Given the concise nature of the notes, formal coding procedures were not applied. Instead, notes were organized and summarized into categories reflecting perceived advantages, limitations, and suggested adaptations of the selected interventions to the Georgian context. Selected illustrative quotations from the discussion notes were used to provide examples of participants’ perspectives. All discussion notes were reviewed in de-identified form. To preserve confidentiality while providing context for quotations, illustrative quotations are presented with participants’ stakeholder group (provider or PLWH), age, and gender only. No names or other potentially identifying information were retained in the research materials or reported in the manuscript.

### Ethical considerations

Ethical approval for this study was obtained from the Institutional Review Board of the National Center for Disease Control and Public Health of Georgia (IRB # 2025−017). All participants provided written informed consent before the session began.

## Results

A total of 22 participants took part in the NGT sessions: 12 HIV care providers and 10 PLWH. The provider group included clinicians, nurses, epidemiologists, and other healthcare professionals involved in the care and support of PLWH, 2 men and 10 women, age range 35–70 years. Among the PLWH participants, 3 were men and 7 were women, age range 34–55 years.

### Selection of the most appropriate intervention format

In the provider NGT session, participants compared session-based, paper-based, and digital intervention formats in terms of their appropriateness for the Georgian HIV care context. Session-based approaches were viewed as potentially useful for older age groups and for building trust through direct interaction, but participants also noted that group-based formats may be uncomfortable for some individuals and that the quality of facilitation would be critical. As one participant noted, *“Older patients may prefer talking to someone directly rather than using a phone or computer”* [52-year-old female, provider]. At the same time, others observed that *“group discussions may not be comfortable for everyone, especially when the topic is very personal”* [35-year-old female, provider]. Paper-based materials were considered easy to distribute and potentially useful for people with limited access to digital resources, but were generally viewed as less effective because participants felt that such materials are often not read carefully and may be too passive to support complex decision-making. One participant noted that *“people often receive brochures but do not actually read them”* [51-year-old male, provider]. Digital interventions received the highest ranking score, compared with session-based and paper-based. Providers emphasized the accessibility, convenience, and appeal of digital tools, particularly for younger users, although they also noted that such tools should avoid overly technical language and may be less suitable for older adults or those with limited digital literacy. As reflected in the discussion notes, digital tools were seen as *“easy to access”* and *“more suitable for younger age groups.”*

In the PLWH NGT session, participants also compared the three intervention formats, but their preferences differed from those of providers. Session-based interventions received the highest ranking score, followed by digital interventions, while paper-based interventions received no support. Participants emphasized the importance of direct communication, trust, and opportunities to hear from others with similar experiences when discussing HIV disclosure. One participant explained that *“it is easier to talk about these issues with someone who understands your situation”* [34-year-old female, PLWH]. Session-based approaches were seen as especially valuable because they allow space for questions, shared experiences, and support during a highly stressful period following diagnosis. By contrast, paper-based materials were viewed as burdensome and unlikely to be read, especially when individuals are emotionally overwhelmed. As one participant noted, *“when someone has just learned about their diagnosis, reading long materials can be very difficult”* [55-year-old male, PLWH]. Digital interventions were recognized as offering privacy, anonymity, and broad accessibility, but some participants expressed doubts about the credibility of digital sources unless references were clearly indicated, noting that *“people need to know where the information comes from in order to trust it”* [48-year-old female, PLWH]. Across the discussion, participants emphasized that no single format would be ideal for everyone and that peer involvement should be an essential component of any intervention. As one participant summarized, *“it is important to hear from people who have already gone through this experience”* [40-year-old female, PLWH]. Ranking scores for intervention format from both groups are summarized in Table 1.

**Table 1:** Ranking scores for intervention format by participant group.

Intervention format	Providers (n = 12)	PLWH (n = 10)
Session-based	13	22
Paper-based	5	0
Digital	18	8

**Note:** Ranking scores represent the sum of scores assigned by participants during the NGT sessions. Higher scores indicate greater perceived appropriateness and feasibility of the intervention format for the Georgian context.

### Selection of the most appropriate intervention and suggestion for adaptation

Among providers, the second ranking exercise focused on the three digital interventions. Two interventions were viewed favorably, but READY received the highest ranking score, narrowly exceeding DISCLOSURE, while “Who, When, How to Share” received substantially lower support. READY was preferred because participants considered it compact, clearly structured, and easier for users to complete in manageable steps. DISCLOSURE was also viewed positively, especially because of its visual and video components, but providers considered READY slightly more practical and adaptable. In the final adaptation discussion, providers emphasized that the selected intervention should address disclosure to different categories of people, including spouses or partners, family members, friends, and healthcare personnel. Particular importance was placed on disclosure to a spouse or sexual partner, which participants linked both to prevention concerns and to legal obligations in Georgia. As one provider noted, *“Disclosure to a spouse or partner is especially important, both for prevention and because there are legal responsibilities involved”* [65-year-old female, provider]. Providers also recommended including HIV-specific content on legal and legislative issues, access to legal consultation, post-exposure issues, patients’ rights, and available support services.

Among PLWH, the second ranking exercise focused on the three session-based interventions. Family disclosure DA intervention received the highest ranking score, while HOP and POP received lower scores. Participants expressed a clear preference for an intervention that begins with individual sessions, particularly during the period immediately following diagnosis, when people may not yet feel ready to discuss disclosure in a group. Individual sessions were viewed as allowing more personalized, confidential, and emotionally responsive support, even though participants acknowledged that such an approach may be more time-consuming. In the adaptation discussion, PLWH emphasized that the first sessions should remain individual, that peer educators should play a central role in delivery, and that the number and duration of sessions should be flexible. Participants explained that immediately after diagnosis many individuals need private time and space to process their situation before engaging in group discussions. As one participant noted, *“At the beginning, it is easier to talk one-on-one. People may not be ready to discuss these issues in a group”* [55-year-old male, PLWH]. They also highlighted the need for guidance on disclosure to family members, friends, and healthcare professionals outside specialized HIV services, such as general practitioners and dentists. As one participant explained, *“Sometimes you do not know whether you should tell a dentist or another doctor about your HIV status, so clear guidance would be helpful”* [43-year-old female, PLWH]. Additional suggested content included HIV-related rights and legal protections, treatment adherence, consequences of not initiating or discontinuing treatment, support services, management of medication confidentiality, and common misconceptions about HIV. Participants also noted that confidentiality can be challenging when family members are unaware of the diagnosis, with one participant explaining that *“some people have to hide their medication at home if their family does not know”* [48-year-old female, PLWH]. Ranking scores for interventions within the preferred format from both groups are summarized in Table 2.

**Table 2:** Ranking scores for interventions within the preferred format by participant group.

Providers (n = 12)	
**Digital Intervention**	**Ranking score**
READY	17
DISCLOSURE	16
“Who, When, How to Share”	3
**PLWH (n = 10)**	
**Session-based intervention**	**Ranking score**
Family disclosure DA intervention	20
HOP	5
POP	5

**Note:** Ranking scores represent the sum of scores assigned by participants during the NGT sessions. Higher scores indicate greater perceived appropriateness and feasibility of the intervention for the Georgian context.

Following the two separate NGT sessions, a joint session was held with representatives from both groups to review the findings and reach consensus on the intervention to be adapted. The facilitator presented the intervention ranking results, the main points raised during discussion, and the intervention adaptation suggestions generated during the provider and PLWH NGT sessions. Participants noted that providers had placed greater emphasis on feasibility, accessibility, and scalability, whereas PLWH had emphasized trust, individualized support, peer involvement, and the emotional realities of disclosure decision-making after diagnosis. During the joint discussion, consensus emerged that the preferences of PLWH should be given priority because they represent the primary target population of the intervention. The group therefore selected the Family disclosure DA intervention as the final intervention for adaptation in the Georgian context. Participants considered this intervention most responsive to the need for individualized, flexible, and supportive decision-making around HIV disclosure.

## Discussion

This study used the NGT to identify and prioritize an intervention to support HIV status disclosure decision-making among PLWH in Georgia. The findings revealed important differences between HIV care providers and PLWH in their preferred intervention formats. Providers tended to prioritize digital approaches, emphasizing accessibility, convenience, and potential scalability within health services. In contrast, PLWH expressed a clear preference for session-based interventions, highlighting the importance of trust, direct communication, and emotional support when navigating complex disclosure decisions. During the joint session, participants agreed that the perspectives of PLWH should guide the final selection, given that they represent the primary target population of the intervention. Overall, the findings underscore that HIV disclosure decision-making is not only an informational process but also a highly personal and relational experience that may require individualized approaches and adaptation of relevant DA interventions.

The differences observed between providers and PLWH in preferred intervention formats likely reflect the distinct roles, priorities, and lived experiences that each group brings to HIV care and decision-making. Providers tended to emphasize considerations related to feasibility, accessibility, and scalability, which are common priorities in clinical and programmatic settings where interventions must be implemented across larger patient populations. In contrast, PLWH highlighted relational and emotional aspects of disclosure decision-making, emphasizing the importance of trust, peer support, and individualized communication. Previous research similarly shows that provider and patient perspectives in HIV care may differ and suggest a gap in provider’s understanding of clients, with providers often focusing on clinical or system-level considerations while PLWH place greater importance on psychosocial support, confidentiality, and the quality of interpersonal interactions within care settings [28–31]. The final decision to prioritize the PLWH-preferred intervention reflects the study’s focus on selecting an intervention that is responsive to the needs of its intended users. At the same time, provider concerns regarding feasibility should inform future adaptation efforts, including consideration of intervention length, staffing requirements, integration into existing HIV services, and opportunities to incorporate scalable components where appropriate.

The strong preference among PLWH for individualized support at the beginning of the disclosure decision-making process likely reflects the emotional and cognitive burden that often follows an HIV diagnosis. Research shows that newly diagnosed people commonly experience high levels of stress, uncertainty, and anxiety, which can make decision-making particularly difficult in the early period after diagnosis [32–34]. In this context, a one-to-one format may feel safer and more manageable than group discussions, especially when individuals are still deciding whether, when, and to whom they may wish to disclose. At the same time, our findings suggest that support should remain flexible rather than strictly standardized. Participants indicated that readiness to engage in individual or group-based support may vary across individuals and may change over time following diagnosis, highlighting the importance of allowing flexibility in both the format and timing of disclosure DA interventions. In the Georgian setting, this need for individualized and phased support may be particularly important given recent evidence of stigma, discrimination, and the need for stronger legal and psychosocial support for PLWH [35].

The fact that PLWH prioritized an individual session-based intervention focused on disclosure to family members highlights the importance they placed on personalized and confidential support in disclosure decision-making. However, participants also made clear that adaptation would be essential. The adaptation priorities identified by PLWH underscore that disclosure decision-support should be grounded not only in general DA principles but also in the social and legal realities of living with HIV in a specific setting. Participants’ emphasis on peer educators is particularly notable, as peer support has been shown to provide social and emotional support, linkage to care and community resources, and flexible ongoing assistance that complements formal health services for PLWH [36]. In the context of disclosure decision-making, peer involvement may be especially valuable because it can increase trust, normalize difficult experiences, and make guidance feel more credible and relevant to real-life situations. This is an important consideration in settings where stigma and fear of negative consequences continue to shape disclosure decisions [2]. At the same time, participants’ recommendations to include information on legal rights, treatment adherence, available support services, medication confidentiality, and disclosure to non-HIV healthcare providers suggest that the intervention cannot simply be transferred unchanged from another setting. Instead, it needs contextual adaptation so that it addresses the practical questions and risks that PLWH actually face in Georgia. This interpretation is consistent with broader evidence showing that culturally and contextually tailored HIV interventions tend to be more acceptable and more responsive to psychosocial needs than non-adapted approaches [37].

This study has several limitations. First, younger PLWH were underrepresented in the NGT sessions. This may be an important limitation because younger PLWH may have different experiences, preferences, and communication patterns related to HIV disclosure, particularly in relation to digital technologies, social media, and peer networks. Future research should aim to include a broader age range of participants to better capture generational differences in disclosure experiences and preferences for decision-support interventions. Second limitation relates to the gender composition of the participant groups. Most HIV care providers participating in the NGT sessions were women, although this generally reflects the gender distribution of the healthcare workforce in Georgia. Women were also overrepresented among PLWH participants. As gender may influence HIV disclosure experiences and preferences for disclosure DA interventions, the perspectives identified in this study may not fully reflect those of men living with HIV. Furthermore, participants were recruited from Tbilisi, and the findings may not fully capture the experiences and preferences of PLWH and providers in other regions of Georgia. Future studies should include more geographically and demographically diverse samples to explore how intervention preferences vary across different population groups and settings. Third, the discussions were documented through field notes rather than full verbatim transcripts, which may have limited the depth of qualitative analysis and the ability to capture the full nuance of participants’ perspectives. In addition, although NGT provides a structured way to generate and prioritize ideas, the method focuses primarily on consensus building and ranking rather than in-depth exploration of experiences, and therefore may not capture the full complexity of participants’ views. Another limitation is that the study was not designed to examine differences in intervention preferences according to participant characteristics such as gender, age, living status, or other socio-demographic factors. Although these characteristics may influence HIV disclosure experiences and attitudes toward disclosure support interventions, the sample size and NGT methodology were not intended to support subgroup analyses. Future research with larger and more diverse samples could explore how preferences for disclosure DA interventions vary across demographic and social groups. Despite these limitations, the structured format of NGT allowed diverse stakeholders to contribute equally and provided a transparent process for identifying priorities for intervention adaptation.

## Conclusion

This study identified and prioritized a disclosure decision-support intervention for adaptation to the Georgian context using a structured NGT process that incorporated perspectives from both HIV care providers and PLWH. The findings highlight the importance of selecting interventions that align with the needs and lived experiences of PLWH, particularly in relation to individualized and context-sensitive support for disclosure decision-making. The selected intervention will serve as the focus of subsequent research. Future studies will adapt this intervention to the Georgian context to assess barriers and facilitators to implementation among both clients and providers, and to identify appropriate strategies to address these barriers, thereby laying the groundwork for pilot testing and evaluating its feasibility and effectiveness in real-world settings. Future research should also examine implementation approaches that balance the preferences of PLWH with the practical constraints and priorities of healthcare providers and service systems.

## Supporting information

S1 File Data representing participant-level ranking scores from nominal group technique sessions (providers and PLWH). (DOCX)

S2 File Data representing qualitative notes from nominal group technique sessions (providers and PLWH). (DOCX)
