# Supplementary material for: Using nominal group technique to select an HIV status disclosure decision aid for adaptation in Georgia
Source: PLoS One. 2026 Jul 16;21(7):e0353949. doi: 10.1371/journal.pone.0353949 (PMC13374881; doi:10.1371/journal.pone.0353949)
Supplement: S1 File — (DOCX) [file pone.0353949.s001.docx]

**Supplementary File 1. Data_Participant-Level Ranking Scores from Nominal Group Technique Sessions (Providers and PLWH)**

**Provider-level ranking of intervention formats**

| **Participant** | **Intervention Format** | | |
| --- | --- | --- | --- |
|  | **Session-based** | **Paper-based** | **Digital** |
| Provider 1 | 0 | 0 | 3 |
| Provider 2 | 0 | 0 | 3 |
| Provider 3 | 1 | 0 | 2 |
| Provider 4 | 1 | 0 | 2 |
| Provider 5 | 1 | 0 | 2 |
| Provider 6 | 0 | 1 | 2 |
| Provider 7 | 2 | 0 | 1 |
| Provider 8 | 2 | 1 | 0 |
| Provider 9 | 1 | 1 | 1 |
| Provider 10 | 2 | 0 | 1 |
| Provider 11 | 1 | 2 | 0 |
| Provider 12 | 2 | 0 | 1 |
| **Total** | **13** | **5** | **18** |

**Provider-level ranking of digital interventions**

| **Participant** | **Intervention Format** | | |
| --- | --- | --- | --- |
|  | **READY** | **DISCLOSURE** | **“Who, When, How to Share”** |
| Provider 1 | 3 | 0 | 0 |
| Provider 2 | 3 | 0 | 0 |
| Provider 3 | 3 | 0 | 0 |
| Provider 4 | 2 | 1 | 0 |
| Provider 5 | 2 | 1 | 0 |
| Provider 6 | 2 | 1 | 0 |
| Provider 7 | 1 | 2 | 0 |
| Provider 8 | 1 | 2 | 0 |
| Provider 9 | 0 | 3 | 0 |
| Provider 10 | 0 | 3 | 0 |
| Provider 11 | 0 | 2 | 1 |
| Provider 12 | 0 | 1 | 2 |
| **Total** | **17** | **16** | **3** |

**PLWH-level ranking of intervention formats**

| **Participant** | **Intervention Format** | | |
| --- | --- | --- | --- |
|  | **Session-based** | **Paper-based** | **Digital** |
| PLWH 1 | 3 | 0 | 0 |
| PLWH 2 | 3 | 0 | 0 |
| PLWH 3 | 3 | 0 | 0 |
| PLWH 4 | 3 | 0 | 0 |
| PLWH 5 | 2 | 0 | 1 |
| PLWH 6 | 2 | 0 | 1 |
| PLWH 7 | 2 | 0 | 1 |
| PLWH 8 | 2 | 0 | 1 |
| PLWH 9 | 1 | 0 | 2 |
| PLWH 10 | 1 | 0 | 2 |
| **Total** | 22 | 0 | 8 |

**PLWH-level ranking of session-based interventions**

| **Participant** | **Intervention Format** | | |
| --- | --- | --- | --- |
|  | **Family disclosure DA intervention** | **HOP** | **POP** |
| PLWH 1 | 3 | 0 | 0 |
| PLWH 2 | 3 | 0 | 0 |
| PLWH 3 | 3 | 0 | 0 |
| PLWH 4 | 3 | 0 | 0 |
| PLWH 5 | 3 | 0 | 0 |
| PLWH 6 | 2 | 1 | 0 |
| PLWH 7 | 2 | 0 | 1 |
| PLWH 8 | 1 | 2 | 0 |
| PLWH 9 | 0 | 1 | 2 |
| PLWH 10 | 0 | 1 | 2 |
| **Total** | **20** | **5** | **5** |
