# Supplementary material for: Using nominal group technique to select an HIV status disclosure decision aid for adaptation in Georgia
Source: PLoS One. 2026 Jul 16;21(7):e0353949. doi: 10.1371/journal.pone.0353949 (PMC13374881; doi:10.1371/journal.pone.0353949)
Supplement: S2 File — (DOCX) [file pone.0353949.s002.docx]

**Supplementary File 2. Data_Qualitative Notes from Nominal Group Technique Sessions (providers and PLWH)**

**HIV Care Providers (n=12)**

**Moderator:**

**Nominal Question 1**
“What are the main advantages and disadvantages of each intervention group (session-based, paper-based and digital) for use in the Georgian HIV care context?”

**Round-robin responses**

**Provider 1:**

“Each format has advantages, but digital interventions appear most appropriate overall. Session-based approaches are useful in specific contexts, while printed materials are the least effective.”

**Provider 2:**

“Session-based interventions have value, particularly for certain populations, older age groups. Printed materials are often not sufficiently engaging. Digital tools should be prioritized, provided they avoid overly technical language.”

**Provider 3:**

“Session-based approaches may be particularly appropriate for older individuals, but group formats can be limiting. Printed materials may provide background information, although engagement is often low. Digital interventions are efficient and scalable.”

**Provider 4:**

“While session-based interventions allow interaction, group discussions may not be comfortable for everyone, especially when the topic is very personal. Printed materials are simple to distribute but I agree, they tend to be passive. Digital tools provide flexibility and accessibility.”

**Provider 5:**

“Session-based interventions require strong facilitation to be effective. Printed materials may be useful in certain cases, but their impact is limited. Digital tools offer structured and accessible content delivery.”

**Provider 6:**

“Session-based approaches can support communication, but may not be acceptable for all patients. Printed materials are accessible but often not actively used. Digital interventions align better with current user behaviors.”

**Provider 7:**

“Session-based interventions are valuable for building trust through direct interaction, particularly for sensitive topics. People often receive brochures but do not actually read them. Digital tools, on the other hand, are easy to access and highly convenient.”

**Provider 8:**

“I agree with earlier points—session-based formats can be really helpful. Printed materials are often overlooked, but could be acceptable for some. Digital tools are more suitable for younger age groups*.”*

**Provider 9:**

“Session-based approaches support engagement. Printed materials lack interactivity. Digital interventions provide broader reach and convenience.”

**Provider 10:**

“Session-based formats are important for trust-building, but not always feasible. Printed materials are not always effective in practice. Digital tools are efficient and accessible.”

**Provider 11:
“Printed materials are relatively easy to distribute and may be useful for individuals with limited access to digital technologies.”**

**Provider 12:**

“I agree with the previous point. Older patients may prefer talking to someone directly rather than using a phone or computer. Printed materials can support access, but they are often underutilized. Digital interventions are more suitable for routine use, especially for younger populations.”

**Moderator:**

**Nominal Question 2**
“What are the main strengths and limitations of each digital intervention (READY, DISCLOSURE, ‘Who, When, How to Share’)?”

**Round-robin responses**

**Provider 1:**

“READY is compact and clearly structured, which makes it easier for users to complete. DISCLOSURE is useful, but slightly more complex. The third intervention is too long.”

**Provider 2:**

“I agree, READY is practical and divided into manageable steps. DISCLOSURE is good, but requires more time. ‘Who, When, How to Share’ seems too extensive.”

**Provider 3:**

“READY’s structure is a strong advantage. DISCLOSURE has valuable content, but is less straightforward. The third option may be difficult to use in practice.”

**Provider 4:**

“READY is user-friendly. DISCLOSURE benefits from visual materials, including videos. The third intervention is too long.”

**Provider 5:**

“I agree, READY is easier to follow. DISCLOSURE is engaging because of its visual components. The third one is too time-consuming.”

**Provider 6:**

“READY is concise and practical. DISCLOSURE is also useful, especially for engagement. The length of ‘Who, When, How to Share’ is definitely a limitation.”

**Provider 7:**

“DISCLOSURE is more engaging due to videos and visuals. READY is also good but simpler. The third intervention is overly complex.”

**Provider 8:**

“I agree, DISCLOSURE has strong visual design. READY is more practical and easier to implement. The third option may overwhelm users.”

**Provider 9:**

“DISCLOSURE is comprehensive and engaging. READY is practical, but DISCLOSURE is richer. The third intervention is not feasible due to length.”

**Provider 10:**

“I agree, DISCLOSURE stands out because of visual content. READY is still very practical. The third option is too demanding.”

**Provider 11:**

“DISCLOSURE is engaging and informative. READY is efficient. ‘Who, When, How to Share’ has relevant content but needs simplification.”

**Provider 12:**

“The third intervention is very comprehensive, but too long for most users. READY is concise. DISCLOSURE is appealing because of visuals.”

**Moderator:**

**Adaptation Question:**

“What adaptations would be necessary to make the selected digital intervention appropriate for PLWH in the Georgian context?”

**Round-robin responses**

**Provider 1:**

“The intervention should clearly address disclosure to different groups like partners, family members, friends, and healthcare providers.”

**Provider 2:**

“I agree. Disclosure to a spouse or partner is especially important, both for prevention and because there are legal responsibilities involved.*”*

**Provider 3:**

“Legal and legislative aspects of disclosure should be included, as patients often have questions about their obligations.”

**Provider 4:**

“There should also be clear information on patients’ rights.”

**Provider 5:**

“I agree, access to legal consultation or support services should be part of the intervention.”

**Provider 6:**

“Post-exposure issues and prevention-related considerations should also be addressed.”

**Provider 7:**

“The intervention should include practical information, not only general guidance.”

**Provider 8:**

“Content should be tailored to the Georgian context.

**Provider 9:**

“Information about available support services should be clearly presented.”

**Provider 10:**

“The intervention should be structured but adaptable to different users.”

**Provider 11:**

“I agree, different users may need different levels of detail.”

**Provider 12:**

“Overall, the intervention should combine structured guidance with context-specific information.”

**PLWH (n=10)**

**Moderator:**

**Nominal Question 1**

“What are the main advantages and disadvantages of each intervention group (session-based, paper-based and digital) for use in the Georgian HIV care context?”

**Round-robin responses**

**PLWH 1:
“Session-based support is very important, it is easier to talk about these issues with someone who understands your situation. Printed materials are not very helpful because people don’t read them. Digital tools can be useful, but they are not enough on their own.”**

**PLWH 2:
“I agree. Talking to someone directly helps a lot, especially when you can ask questions. Printed materials are difficult to focus on. Digital tools can be good because they are private.”**

**PLWH 3:
“Session-based approaches are helpful because you can hear other people’s experiences, especially if it’s peer. Printed materials are often ignored. Digital tools might also be useful, especially if they are easy to use.”**

**PLWH 4:
“Session-based support is best, but timing is important. Printed materials are difficult, when someone has just learned about their diagnosis, reading long materials can be very difficult. Digital tools can help if they are simple.”**

**PLWH 5:
“I agree, session-based support is most helpful. Printed materials are usually not read. Digital tools are useful, but not always trusted.”**

**PLWH 6:
“I agree with others, session-based support is the most important, especially to hear from peers. Printed materials don’t really work. Digital tools can help, but it is important to hear from people who have already gone through this experience.”**

**PLWH 7:
“Session-based approaches are important for support. Printed materials are not very useful. For digital tools, people need to know where the information comes from in order to trust it*.*”**

**PLWH 8:
“Session-based formats help because you can talk and understand better, you can ask questions. Printed materials are too much to read. Digital tools might be good because of their privacy.”**

**PLWH 9:**

**“At the beginning it’s very hard. Session-based support is important later, when you are more ready. Printed materials are not effective. Digital tools can give you lot of information information privately.”**

**PLWH 10:**

**“Sometimes people are not ready to join sessions right away. Printed materials are not practical in that situation. I would prefer digital tools, but only if they are trustworthy.”**

**Moderator:**

**Nominal Question 2**

“What are the main strengths and limitations of each session-based intervention (Family Disclosure Decision-Making Intervention, HOP, POP)?”

**Round-robin responses**

**PLWH 1:**

“Individual sessions are the most important—you can talk about your own situation in private. Group sessions like HOP or POP are harder at the beginning.”

**PLWH 2:**

“I agree, after diagnosis, you are not ready to talk in a group. Individual sessions feel safer.”

**PLWH 3:**

“With individual sessions, the support is more personal. Group sessions can help later, but not at first.”

**PLWH 4:**

“At the beginning, you need time and privacy. Group discussions may come later.”

**PLWH 5:**

“Individual sessions are more comfortable. In groups, some people may not want to speak.”

**PLWH 6:**

“Individual sessions are most helpful, but HOP can also help you hear others’ experiences. POP seems similar.”

**PLWH 7:**

“I prefer individual sessions, but group formats like POP may be useful later.”

**PLWH 8:**

“HOP is helpful because you hear different experiences. Individual sessions are also important, especially at the beginning.”

**PLWH 9:**

“Group sessions like POP help you understand others, but individual sessions are still important.”

**PLWH 10:**

“I agree, group sessions can be supportive, but at the beginning people need individual support.”

**Moderator:**

**Adaptation Question:**

“What adaptations would be necessary to make the selected session-based intervention appropriate for PLWH in the Georgian context?”

**Round-robin responses**

**PLWH 1:**

“At the beginning, it is easier to talk one-on-one. People may not be ready to discuss these issues in a group. So, you can start with individual and then continue with group sessions”

**PLWH 2:**

“I agree, first sessions should be individual, especially right after diagnosis.”

**PLWH 3:**

“I also agree. People need time to process everything before joining group discussions. Also, there should be information about rights and legal protections.”

**PLWH 4:**

“Individual sessions feel more private and safe at the beginning.”

**PLWH 5:**

“I agree, later, group sessions can be helpful, but not at the start.”

**PLWH 6:**

“Peer educators should be involved in delivery of the intervention; they understand the situation better. Also information should be about treatment, for example what happens if you stop or don’t start treatment.”

**PLWH 7:**

“I agree, people trust peers who have gone through the same experience, so they should be involved in intervention delivery.”

**PLWH 8:**

“The number and length of sessions should be flexible. Support services should be clearly explained.”

**PLWH 9:**

“Sometimes you do not know whether you should tell a dentist or another doctor about your HIV status, so clear guidance would be helpful. Also, some people have to hide their medication at home if their family does not know, this should also be addressed by the intervention”

**PLWH 10:**

“I agree, there should also be information about family and friends, not only doctors or vice versa.”
